# Pretreatment with Low-Dose Esketamine for Reduction of Propofol Injection Pain: A Randomized Controlled Trial

**DOI:** 10.1155/2022/4289905

**Published:** 2022-08-02

**Authors:** Danyun Fu, Dingding Wang, Wenxian Li, Yuan Han, Jie Jia

**Affiliations:** Department of Anesthesiology, Eye & ENT Hospital of Fudan University, Xuhui, Shanghai 200031, China

## Abstract

**Background:**

Propofol-induced injection pain is a common adverse effect during the induction of general anesthesia. The purpose of this study is to investigate the effect of low-dose esketamine in preventing propofol injection pain.

**Methods:**

In this double-blind, randomized, controlled trial, patients scheduled for elective ear surgery under general anesthesia received either normal saline (NS), or 40 mg lidocaine, or 0.15 mg/kg esketamine 30 seconds before manual injection of propofol. The primary outcome of this study was the incidence of propofol injection pain. The secondary outcomes included injection pain score, vital signs, total dosage of vasoactive drugs used within 5 minutes after induction, and adverse events related to drugs.

**Results:**

A total of 105 patients were included. Compared with the NS group (67%), pretreatment with esketamine and lidocaine significantly reduced the incidence of injection pain to 29% and 33%, respectively (both *P* < 0.05); however, no significant difference was found between the esketamine and lidocaine groups. The median of injection pain score was significantly lower in the esketamine and lidocaine groups (both median (interquartile range) = 0 (0–1)) than that in the NS group (1 (0–2); *P* < 0.05). In addition, compared with the NS and lidocaine groups, preinjection esketamine provided more stable hemodynamic parameters within 5 minutes after induction (*P* < 0.05). No statistical difference was found in adverse events among the three groups.

**Conclusions:**

Pretreatment with a low-dose esketamine can not only reduce the incidence of propofol injection pain but also provide a more stable circulation in patients after anesthesia induction. This convenient, well-tolerated, and economic treatment appears as an option to be routinely applied in clinic practice. *Clinical Trial Registration.* This trial is registered with https://www.chictr.org.cn/showproj.aspx?proj=136690 (the number for the trial registration isChiCTR2100052742).

## 1. Introduction

Propofol is widely used in the induction of general anesthesia due to its rapid onset and early recovery without accumulation; however, pain is a common adverse effect during propofol injection [1]. The incidence of injection pain can be as high as 80%–90% in an untreated patient [2]. Hemodynamic fluctuation caused by injection pain may trigger myocardial ischemia in high-risk patients [3]. Thus, it is essential to minimize injection pain to avoid such severe adverse events and to improve patients' comfort of anesthesia.

Many strategies have been adopted to prevent injection pain [2, 4–6]. Among which, choosing an antecubital vein and preinjection with lidocaine together with the venous occlusion technique are the two most effective interventions [7]. However, the venous occlusion technique is relatively difficult to be performed. An appropriate dose of lidocaine only prevented injection pain in 50% patients [5]. Nowadays, propofol injection pain still cannot be completely abolished.

From a clinical point of view, pharmacological interventions, which only require drug injection as an adjunctive treatment prior to propofol, are more convenient and feasible to be implemented than the venous occlusion technique. Ketamine exerts its sedative and analgesic effects through activating N-methyl-D-aspartate (NMDA) receptor. Previous studies have reported that a small-dose intravenous ketamine could effectively reduce propofol injection pain in adults and children to different degrees [8–10]; however, the use of ketamine was excluded in China. Esketamine, a right-lateral dismantled fission of ketamine, was included in China in 2020 and can be a more effective alternative instead of ketamine. Compared with ketamine, esketamine has many great advantages including a stronger analgesic effect and a higher clearance rate in in vivo with fewer adverse events such as secretion and psychotomimetic side effects [11, 12]. However, the exact dosage of esketamine to prevent injection pain remains unknown.

Considering the advantages of pharmacological intervention in the clinical setting, we designed this trial to compare the effect of a low-dose esketamine with normal saline (NS) and lidocaine in preventing propofol injection pain.

## 2. Methods

### 2.1. Study Participants

This study was conducted as a randomized, double-blinded, controlled trial at Shanghai Eye and Ear, Nose, and Throat (ENT) Hospital of Fudan University. Ethical approval for this study (ethical committee no. 2020130) was provided by the Ethical Committee of Shanghai Eye and ENT Hospital of Fudan University (floor 3, building 10, no. 83 Fenyang road, Xuhui district, Shanghai, China) on December 22, 2020. The experiment was conducted with the patients' understanding and consents. The trial was registered with China Clinical Research Information Service (registration no. ChiCTR2100052742). The authors prepared this study in accordance with the Consolidated Standards of Reporting Trials (CONSORT) guidelines.

Patients aged 18–65 years, with an American Society of Anesthesiologists (ASA) grade I or II, scheduled to undergo elective ear surgery were included. Patients were excluded if they were allergic to experimental drugs, had a serious risk of increased blood pressure (quiet blood pressure >170/100 mmHg) or intracranial pressure, had a history of hyperthyroidism, serious functional insufficiency of important organs, significant ischemic heart disease, sinus tachycardia, abuse of alcohol, analgesia, or sedative antidepressant.

### 2.2. Randomization and Blinding

Eligible adult patients were randomly assigned into three groups with 35 patients in each group by computer-generated allocation. A specific anesthesia nurse who did not participate in this study generated a random allocation sequence and enrolled participants. The patients in group NS, group lidocaine, and group esketamine received pretreatment with NS, 40 mg lidocaine, and 0.15 mg/kg esketamine (Hengrui Medicine Co., Ltd., Jiangsu Province, China), respectively. The results of randomization were sealed in a closed envelope until the day of surgery. The patients and the participating anesthesiologists were both blinded to the results of randomization. The drugs were prepared in a 20 ml syringe with either 20 ml of NS or 40 mg lidocaine, or 0.15 mg/kg esketamine, by a specific anesthesia nurse who did not participate in this study.

### 2.3. Anesthesia Protocol

On arrival in the operating room, the patients received routine monitoring, including electrocardiography, noninvasive blood pressure, and pulse oxygen saturation (SPO_2_). Subsequently, a 20 G intravenous cannula was inserted at the hand dorsum. After preoxygenation, the patients were pretreated with different agents intravenously 30 seconds before injection of 1% propofol medium-chain triglyceride (MCT) and long-chain triglyceride (LCT) emulsion (Fresenius Kabi GmbH., Austria). After pretreating the agents, propofol were manually injected 2 mg/kg (the dosage was based on lean body weight for obese patients) over 15 seconds. The patients were asked the degree of pain at 5 seconds after injection, and the researchers observed facial expressions and body movements and recorded pain scores. All of them were in deep sedation and unable to give a clear response. Then, the patients were injected with sufentanil 0.1 *μ*g/kg and rocuronium 0.6 mg/kg for the induction of general anesthesia, followed by laryngeal mask (LMA) intubation. Dorasetron 0.35 mg/kg was administered 30 minutes before the end of surgery. After the operation, patients were transferred to the resuscitation room until fully recovered.

### 2.4. Study Outcomes

The primary outcome of the study was the incidence of propofol-induced injection pain. The secondary outcomes included injection pain score, vital signs, and cumulative dosages of vasoactive drugs within 5 minutes after induction. Each patient was carefully evaluated for esketamine' adverse events including muscle tremors, rash, postoperative nausea and vomiting, delirium, psychotomimetic, dizziness, and cardiovascular adverse events within 24 hours after injection. Each outcome was evaluated by an anesthesiologist who was blinded to the experimental groups.

Injection pain score was assessed during propofol injection by using a four-point scale as follows: 0 = no pain with negative response to questioning, 1 = mild pain without any behavioral sign, 2 = moderate pain with definite response to questioning accompanied by a behavioral sign, and 3 = severe pain associated with strong vocal response, facial grimacing, withdrawal movement of forearm, or tears [13]. Hemodynamic variables include heart rate (HR) and mean arterial blood (MAP) at the time of waking state (T0), before laryngeal mask placement (T1), immediately after implantation (T2), 3 minutes after implantation (T3), and 5 minutes after implantation (T4). If hypotension occurred (systolic blood pressure (SBP) decreased below 80 mmHg or lower than 30% from the patient's baseline value), ephedrine 6 mg or phenylephrine 0.1 mg in the bolus was administered to the patient intravenously.

### 2.5. Sample Size Calculation

The incidence of propofol-induced pain in patients pretreated with NS was approximately 84% in patients based on previous studies [8, 10]. We expected an incidence of no pain on propofol injection at least 25% reduction in the esketamine treatment group. To achieve a discriminating power of 80% with a 2-sided alpha level of 5%, a sample size of 30 patients in each group was sufficient. Assuming the likelihood of 15% patients dropping out, the sample size was increased to 35 patients per group.

### 2.6. Statistical Analyses

Continuous variables were analyzed by the one-way analysis of variance (ANOVA) test followed by the Dunnett test or Kruskal–Wallis test followed by the Mann–Whitney *U* test. Categorical variables were analyzed by the chi-square test. To obtain a 95% confidence interval (CI), a Bonferroni correction was used to adjust the type I error rate for multiple comparisons. Continuous variables were presented as median (25th and 75th percentiles) or mean ± standard deviation (SD). Categorical variables were presented as number of patients and percentages. All statistical tests are 2-tailed; the corrected differences were considered significant at *P* < 0.05.

## 3. Results

### 3.1. Study Population

**Figure 1:**
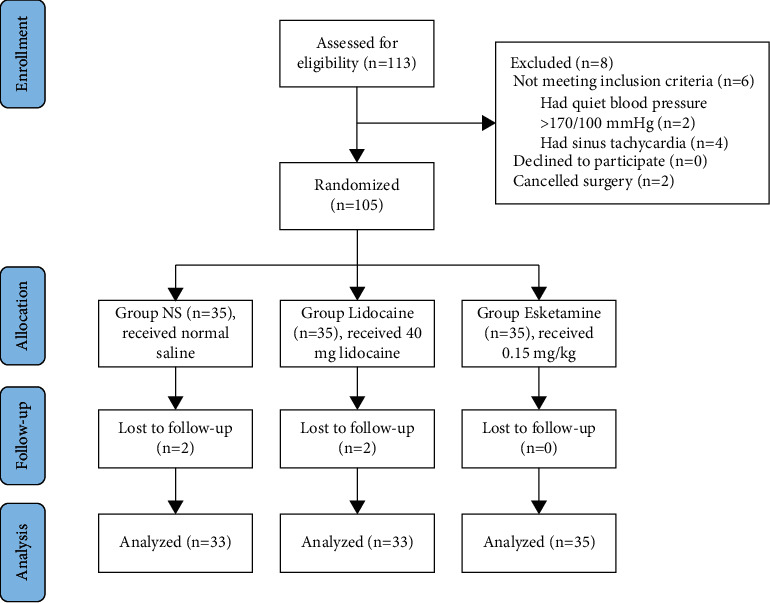
CONSORT flow of clinical procedures for the study. NS, normal saline.

During the period from November 4, 2021, and February 4, 2022, a total of 105 patients were screened for eligibility and 101 patients were ultimately enrolled and analyzed (Figure 1). In the NS and lidocaine groups, 4 subjects were excluded from the study due to lost to follow-up. In the end, 33 subjects were available for analysis in the NS and lidocaine groups, respectively. In the esketamine group, all 35 patients were included in the final analysis (Figure 1). There was no statistical difference in the demographic data among the three groups regarding gender, age, height, weight, body mass index (BMI), ASA grade, proportion of hypertension, duration of operation and recovery, and fluid infusion within 5 minutes after induction (Table 1).

### 3.2. Incidence of Propofol-Induced Injection Pain

**Figure 2:**
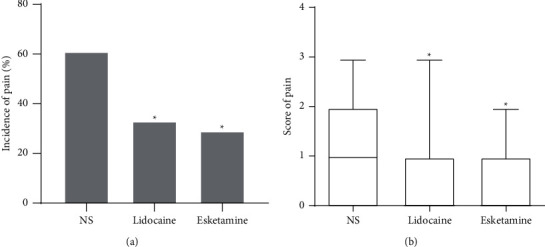
(a) Incidence of injection pain among the three groups. (b) Median injection pain score among the three groups. NS, normal saline. ^*∗*^*P* < 0.05, compared with group NS.

The incidences of propofol injection pain (pain score of 1 or more) are shown in Figure 2(a). 29% patients suffered injection pain in the esketamine group and 33% in the lidocaine group compared with 67% in the NS group (both *P* < 0.05). However, the number of patients experiencing injection pain was not different between group esketamine and group lidocaine, suggesting that esketamine is just as effective as lidocaine.

The median of injection pain score in the esketamine and lidocaine groups was both 0 (0-1), which was significantly lower than that in the NS group (1 (0–2); *P* < 0.05) (Figure 2(b)). The incidences of severity of pain (mild/moderate/severe) were lower in the esketamine and lidocaine groups in comparison with those in the NS group, but these were not statistically significant (Table 2).

### 3.3. Hemodynamic Parameters and Vasoactive Drugs

**Figure 3:**
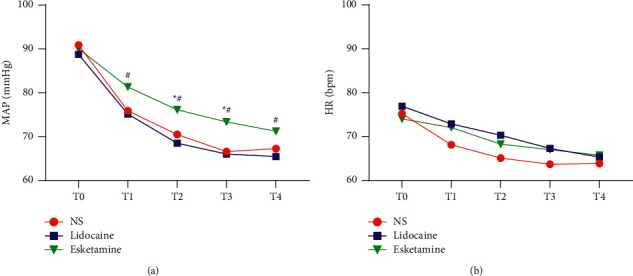
Changes in the hemodynamic parameters among the three groups. (a) Changes in MAP among the three groups. (b) Changes in HR among the three groups. NS, normal saline; T0, at the time of waking state; T1, before laryngeal mask placement; T2, immediately after implantation; T3, 3 minutes after implantation; T4, 5 minutes after implantation. ^*∗*^*P* < 0.05, compared with group NS. ^#^*P* < 0.05, compared with group lidocaine.

The hemodynamic data measured during 5 minutes within induction are shown in Figure 3. Esketamine significantly prevented the MAP reduction from T1 to T4 compared with lidocaine (Figure 3(a), *P* < 0.05), but had no significant effect on HR (Figure 3(b)). Compared with esketamine, the MAP was significantly decreased in the NS group from T2 to T3 (*P* < 0.05), accompanied with a slight increase in T4, which may be due to the injection of vasoactive drugs (Figure 3(a)). In addition, the MAP changes between T0 and T2, T0, and T3 were significantly smaller in group esketamine than those in group NS (Table 3). Anesthetic protocol and volume of infused fluid within 5 minutes after induction were similar in each group (Table 1).

The cumulative dosage of phenylephrine during 5 minutes after induction was lower in the esketamine group compared with that in the NS and lidocaine groups; however, this did not reach a statistical significance (Table 4).

### 3.4. Adverse Events Related to Drugs among Different Groups

The distribution and incidence of adverse events in each group are given in Table 4. The proportion of patients experiencing nausea and vomiting was higher in the esketamine group than in other groups; however, this did not reach a statistical significance (Table 4). No psychotomimetic symptoms and major cardiovascular adverse events were recorded.

## 4. Discussion

In this study, we found that a low-dose esketamine at 0.15 mg/kg was as effective as lidocaine in attenuating propofol injection pain compared with NS. However, esketamine was more beneficial in providing a stable hemodynamic profile than lidocaine and NS during induction.

A previous study reported that ketamine reduced propofol injection pain from 84.8% to 17% at a dosage of 0.3 mg/kg [10]. Considering that the 0.3 mg/kg dosage of ketamine was effective and esketamine has an approximately two-fold higher sedation and analgesia effect than that in ketamine [12], we therefore applied esketamine at a dosage of 0.15 mg/kg in a preliminary experiment. In this study, we found that pretreatment with 0.15 mg/kg esketamine reduced the propofol injection pain incidence to 29%. In addition, the treatment effect was similar in alleviating injection pain between esketamine and lidocaine pretreatment. It added more information about the dosage of esketamine for reducing this type of pain.

The major mechanism responsible for pretreatment with ketamine 30 seconds before propofol may be through peripheral local anesthetic action in the vascular endothelium, rather than a central analgesic effect [9]. The exact mechanism of esketamine may be similar to that of ketamine. Therefore, we considered that esketamine ameliorated propofol injection pain mainly through a peripheral pathway.

Previous studies have shown that a combination of propofol and ketamine is more beneficial for maintaining hemodynamic stability due to the cardiostimulant effects of ketamine balancing the cardiodepressant effects of propofol [14–16]. Considering arterial pressure peaking between 2 and 5 min after injection of ketamine [17] and a higher clearance of esketamine [12], we observed the MAP during 5 min after anesthesia induction. In our study, the MAP was more stable in patients receiving esketamine compared with NS and lidocaine, which was consistent with previous studies regarding ketamine [15].

It is well known that the use of ketamine as a single sedative agent has been limited by its psychotomimetic effects and other side effects [18]. Our data showed that no significant differences were found in the adverse events among the three groups, which may be possibly due to the low dose of esketamine. This study also found that pretreatment with esketamine did not affect the recovery time. These results demonstrated that a low-dose esketamine was relatively safe as an adjunct to general induction with propofol.

In our study, pretreatment with esketamine not only reduced the incidence of pain injection but also provided a stable hemodynamic profile after induction. From a clinical point of view, premedication with esketamine is easy and convenient to be implemented as well as economic wise in daily practice. The results of this study can be clinically applied in general.

A limitation of our study is that the propofol injection cannot be eliminated completely. An optimal dosage of esketamine for propofol injection pain should be further examined. Furthermore, this study was only conducted at a single center. In the future, we will coordinate with different hospitals to evaluate the effects of esketamine on propofol-induced injection pain.

## 5. Conclusion

The present study showed that pretreatment with low-dose esketamine at 0.15 mg/kg can relieve propofol-induced injection pain effectively and safely. Furthermore, 0.15 mg/kg esketamine can also provide more stable hemodynamic parameters during induction. Conclusively, pretreatment with a low-dose esketamine appears as a convenient, well-tolerated, and economical option used during the induction of anesthesia. The results of our study provide evidence for improving clinical practice with a positive impact on patient care.

## Figures and Tables

**Table 1:** Baseline patient characteristics.

	NS (*n* = 33)	Lidocaine (*n* = 33)	Esketamine (*n* = 35)
Gender			
Male	11 (33.3)	19 (57.6)	17 (48.6)
Female	22 (66.7)	14 (42.4)	18 (51.4)
Age (year), mean ± SD	45 ± 11	43 ± 13	42 ± 13
Height (kg), mean ± SD	164 ± 8	166 ± 8	164 ± 7
Weight (kg), mean ± SD	62 ± 11	65 ± 13	62 ± 13
BMI, mean (SD)	23 ± 3	23 ± 4	23 ± 4
ASA I/II grade, *n* (%)			
I	24 (72.7)	28 (84.8)	29 (82.9)
II	9 (27.3)	5 (15.2)	6 (17.1)
Hypertension, *n* (%)			
Yes	5 (15.2)	4 (12.1)	3 (8.6)
No	28 (84.8)	29 (87.9)	32 (91.4)
Duration of operation (min), mean ± SD	55 ± 23	69 ± 37	68 ± 35
Duration of recovery (min), mean ± SD	36 ± 7	39 ± 14	40 ± 18
Fluid infusion within 5 minutes after induction (ml), mean ± SD	140 ± 45	135 ± 35	130 ± 42

Gender, ASA grade, and hypertension are expressed as the number (%), while other values are expressed as mean ± SD. ASA, American Society of Anesthesiologist; NS, normal saline.

**Table 2:** Number of patients experiencing propofol injection pain among three groups.

Severity of pain, *n* (%)	None (0 point)	Mild (1 point)	Moderate (2 points)	Severe (3 points)
NS (*n* = 33)	11 (33.3)	13 (39.4)	6 (18.2)	3 (9.1)
Lidocaine (*n* = 33)	23 (69.7)^*∗*^	6 (18.2)	3 (9.1)	1 (3.0)
Esketamine (*n* = 35)	25 (71.4)^*∗*^	8 (22.9)	2 (5.7)	0 (0)

Data are presented as the number of patients (%). NS, normal saline. ^*∗*^*P* < 0.05, compared with group NS.

**Table 3:** HR and MAP changes between baseline and different time points.

		NS (*n* = 33)	Lidocaine (*n* = 33)	Esketamine (*n* = 35)
HR (beats per minute)	T0-T1	7.12 ± 10.97	4.00 ± 9.88	1.94 ± 7.69
	T0–T2	10.15 ± 11.58	6.61 ± 9.90	5.74 ± 8.15
	T0–T3	11.58 ± 12.84	9.58 ± 9.57	6.97 ± 8.75
	T0–T4	11.39 ± 11.82	11.58 ± 11.27	8.26 ± 9.89

MAP (mmHg)	T0-T1	15.00 ± 14.5	13.61 ± 9.49	8.74 ± 12.18
	T0–T2	20.39 ± 13.21	20.27 ± 9.56	13.94 ± 11.88^*∗*^
	T0–T3	24.30 ± 14.01	22.73 ± 11.08	16.71 ± 11.13^*∗*^
	T0–T4	23.64 ± 13.38	23.30 ± 11.61	18.86 ± 10.64

Data are shown as means ± SD. NS, normal saline; T0, at the time of waking state; T1, before laryngeal mask placement; T2, immediately after implantation; T3, 3 minutes after implantation; T4, 5 minutes after implantation. ^*∗*^*P* < 0.05, compared with group NS.

**Table 4:** Adverse events and vasoactive drug dosage among different groups (*n* (%)).

Group	NS (*n* = 33)	Lidocaine (*n* = 33)	Esketamine (*n* = 35)
Adverse events			
Tremors	0 (0%)	1 (3%)	0 (0%)
Rash	0 (0%)	0 (0%)	2 (6%)
Nausea and vomiting	3 (9%)	5 (15%)	7 (20%)
Delirium	0 (0%)	0 (0%)	1 (3%)
Psychotomimetic symptom	0 (0%)	0 (0%)	0 (0%)
Dizziness	3 (9%)	3 (9%)	5 (15%)
Cardiovascular adverse events	0 (0%)	0 (0%)	0 (0%)
Vasoactive drugs			
Ephedrine (mg)	2.00 ± 3.65	1.09 ± 2.35	1.20 ± 2.54
Phenylephrine (*μ*g)	51.52 ± 143.9	54.55 ± 143.8	12.86 ± 53.33

NS, normal saline; T0, at the time of waking state; T1, before laryngeal mask placement; T2, immediately after implantation; T3, 3 minutes after implantation; T4, 5 minutes after implantation.

## Data Availability

The data used to support this study are available from the corresponding author upon request.
